# Novel Therapies for Myocardial Irritability following Extreme Hydroxychloroquine Toxicity

**DOI:** 10.1155/2015/692948

**Published:** 2015-08-17

**Authors:** Paul B. McBeth, Perseus I. Missirlis, Harry Brar, Vinay Dhingra

**Affiliations:** Division of Critical Care Medicine, University of British Columbia, Vancouver General Hospital, Vancouver, BC, Canada V5Z 1M9

## Abstract

*Introduction*. Hydroxychloroquine (HCQ) overdose is rare and potentially deadly when consumed in large doses. Management of severe HCQ toxicity is limited and infrequently reported. This report presents the case of a massive ingestion of HCQ. *Case Report*. A 23-year-old female presents following an intentional ingestion of approximately 40 g of HCQ. Within six hours after ingestion, she developed severe hemodynamic instability resulting from myocardial irritability with frequent ventricular ectopic activity leading to runs of polymorphic ventricular tachycardia (PMVT) and ventricular fibrillation (VF) requiring multiple defibrillations. Additional treatments included intravenous diazepam, epinephrine, norepinephrine, sodium bicarbonate, and magnesium sulfate. Despite the ongoing hemodynamic instability, the patient was also treated with Intralipid (ILE) and received hemodialysis. Improvements in her hemodynamics were observed after 18 hours. She survived her massive overdose of HCQ. *Conclusion*. HCQ poisoning is rare but serious because of its rapid progression to life-threatening symptoms. Hemodynamic support, gastric decontamination, electrolyte monitoring and replacement, and management of arrhythmias are the mainstays of treatment. The combined role of dialysis and ILE in the setting of massive HCQ overdose may improve outcomes.

## 1. Introduction

Hydroxychloroquine (HCQ) overdose is rare and often lethal when ingested in large doses. There exists a paucity of data on management of HCQ toxicity as it is infrequently reported. The majority of treatment recommendations are extrapolated from chloroquine (CQ) poising [1, 2]. Current management strategies are targeted at myocardial stabilization, hemodynamic support, electrolyte correction, and decontamination [1, 2]. We herein report a unique case of a massive (~40 g) ingestion of HCQ complicated by coma, hemodynamic instability, and respiratory failure treated with mechanical ventilation, vasopressor, and inotropic support, as well as hemodialysis and intravenous lipid emulsion (Intralipid) (ILE).

## 2. Case Presentation

### 2.1. Clinical Presentation

**Figure 1:**
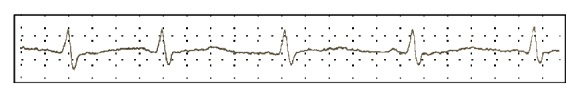
Presenting ECG tracing.

A 23-year-old female with a past medical history of depression, borderline personality disorder, obsessive compulsive disorder, psychosis NOS, and congenital hydrocephalus with VP shunt presents following ingestion of approximately 40 g of HCQ. She had been using HCQ for the treatment of pruritus. She was also being treated with risperidone, clonazepam, and zopiclone for her psychiatric disorder and fluconazole at the time of presentation for the treatment of a vaginal yeast infection. At presentation, she had a Glasgow Coma Score (GCS): 15/15, temperature: 37.1°C, blood pressure (BP): 92/60 mmHg, pulse rate: 65/min, respiratory rate: 16/min, blood glucose: 3.9 mmol/L, and serum potassium: 3.0 mmol/L. The initial blood gas demonstrated a metabolic acidosis and respiratory alkalosis: pH: 7.33, pCO_2_: 16, pO_2_: 70, and bicarbonate: 8. Electrocardiograph performed on arrival demonstrated sinus rhythm with a widened QRS (140 ms) and QT interval (QTc 576 ms). See Figure 1. Her urine toxicology was negative for salicylates, ethanol, acetaminophen, benzodiazepines, cannabinoid, cocaine, opiates, and amphetamines. The presenting renal and hepatic laboratory values are as follows: creatinine (Cr): 133 *μ*mol/L; albumin: 39 g/L; lipase: 113 units/L; gamma-glutamyl transpeptidase (GGT): 30 units/L; alkaline phosphatase: 55 units/L; total bilirubin: 3 *μ*mol/L; direct bilirubin: 1 *μ*mol/L; alanine aminotransferase (ALT): 23 units/L; aspartate aminotransferase (AST): 14 units/L.

### 2.2. Resuscitation and Management

Within three hours of her overdose, the patient became obtunded requiring intubation and mechanical ventilation. Hypotension was treated with crystalloid and vasopressor support. Gastric lavage and activated charcoal for decontamination were provided. Within six hours of her overdose, she demonstrated severe hemodynamic instability resulting from myocardial irritability with frequent ventricular ectopic activity leading to runs of polymorphic ventricular tachycardia (PMVT) and ventricular fibrillation (VF) requiring 18 defibrillations with 200J with biphasic defibrillator over the first 18 hours after ingestion. Given her protracted cardiac instability, additional treatments including intravenous diazepam 60 mg followed by infusion of 6 mg/hr for calcium channel stabilization (total of 82 mg given), epinephrine 20 mcg/min, norepinephrine 30 mcg/min, sodium bicarbonate 30 mmol/hr, and magnesium sulfate 1 g/hr infusions were also initiated. The serum potassium three hours after the overdose was 1.5 mmol/L. In total, 380 mmol of potassium chloride was given over the first 18 hours administered in 20 mmol boluses. She also received intravenous sodium bicarbonate for alkalization. To treat refractory ventricular ectopy, a bolus dose of 1.5 mL/kg of 20% Intralipid ILE was initially given followed by a high dose infusion over approximately 30 minutes. A total of 500 mL of ILE was given. No further infusion of ILE was given after this initial dose. Vascular access for dialysis was established immediately after the bolus dose and thus dialysis overlapped briefly with the infusion of ILE. The runs of PMVT and VF were felt to be secondary to a preexisting channelopathy likely potentiated by fluconazole and risperidone. Ventricular pacing was reviewed but not considered indicated. The patient was started on piperacillin/tazobactam for possible aspiration pneumonia. Echocardiographic imaging revealed normal global ventricular contractility with an ejection fraction of 60%.

The patient was started on intermittent hemodialysis (IHD) in an attempt to reduce HCQ levels despite a paucity of literature regarding its use. The pharmacokinetics suggests that much of the drug is not accessible to the dialyzer, but given the severity of the overdose and the impact of hemodynamics even a small amount removed was considered favorable. Plasma HCQ level was 6425 mol/L 12 hours after ingestion. A five-hour run of hemodialysis was initiated with end points being either improved hemodynamics or of significant decrease in blood levels. A F1000 Dialyzer was used with a dialysate flow rate of 800 cc/hr. In total, 135 L of blood was processed. As noted above, concurrent with the dialysis run, an ILE infusion was administered in an effort to create a “lipid sink” to sequester lipophilic HCQ. At 31 hours after ingestion, the plasma HCQ level was 4328 mol/L.

Improvements in her hemodynamics were observed after administration of dialysis and ILE. She was subsequently weaned from the ventilator and extubated on day 3 and discharged from ICU on postadmission day six.

## 3. Discussion

Hydroxychloroquine is sold under the trade name Plaquenil and is an aminoquinoline derivative used in the prophylaxis and treatment of malaria. It is also used as an anti-inflammatory agent in rheumatoid arthritis and complications of lupus and connective tissue disorders with an off label use in the treatment of urticaria. Hydroxychloroquine is highly toxic in overdose resulting in rapid onset of hypotension, ventricular dysrhythmias, and cardiac arrest resulting in death [1–3]. Seizures, coma, and respiratory arrest can occur in patients with severe toxicity [3].

Descriptions of HCQ overdose are limited to case reports in the literature. Given this paucity of data, the current emergency treatment is modeled on the experience of CQ overdose. The mortality rate of a CQ overdose for adults is between 10 and 30% [1]. Unlike a CQ overdose, there is no established lethal or toxic dose of HCQ. Treatments are targeted at myocardial stabilization, hemodynamic support, electrolyte correction, decontamination, and prevention of seizures. At present, there is limited data regarding the use of dialysis and to our knowledge there is no description of the use of ILE in combination with dialysis.

Hydroxychloroquine has a dose-related cardiac sodium and potassium channel blocking effect resulting in delayed repolarization and slow intraventricular conduction. This results in bradycardia, hypotension, ventricular dysrhythmias, widened QRS, and prolonged QT interval [4]. Hypokalemia appears to be due to intracellular movement of potassium via a direct effect on cell membrane.

Hydroxychloroquine is rapidly absorbed following ingestion. Peak plasma levels of HCQ occurred 2–4.5 hours after ingestion. In overdose, onset of symptoms usually occurs within 30 minutes. It is highly tissue bound with a large volume of distribution. Distributional half-life is 15–30 hours [5]. Elimination half-life ranges from 4 to 40 days. Death from cardiorespiratory arrest or refractory shock often occurs within 3 hours after ingestion. ECG changes include QRS widening and QT prolongation [3, 6]. Common dysrhythmias include ventricular ectopic beats, ventricular tachycardia, ventricular fibrillation, and torsades de pointes.

Patients presenting to health care facilities less than one-hour after ingestion should be considered for gastric decontamination with gastric lavage and activated charcoal. The use of multiple-dose activated charcoal should be considered in severe HCQ poisoning [7, 8].

Profound hypokalemia is a known effect of HCQ poisoning and appears to correlate with toxicity. The mechanism is related to reduced potassium efflux from the blockade of membrane channels. It is unknown whether HCQ causes direct cardiotoxicity or if it is partly due to the hypokalemia. ECG findings include prolonged QT and QRS intervals leading to life-threatening ventricular arrhythmias and cardiac arrest [3, 6]. Aggressive potassium replacement is required; however, monitoring for rebound hyperkalemia [9–11] with resultant dysrhythmias is important. Diazepam infusion (1-2 mg/kg IV over 30 minutes) has been suggested to modify cardiac toxicity and improve survival based on animal experimental data [4, 12]. This has also been demonstrated in several retrospective studies in patients with acute HCQ toxicity [12–14]. The chronic use of HCQ may lead to QT prolongation [15]. Physicians prescribing HCQ to patients for extended use should consider monitoring patients for cardiac arrhythmias.

The clinical value of hemodialysis and peritoneal dialysis has not been established for the overdose of HCQ. The apparent volume of distribution for HCQ is approximately 600 L/kg. This implies that the drug rapidly becomes unavailable for removal after ingestion [1]. The extensive sequestration of HCQ by tissues limits effectiveness of hemodialysis. Despite the lack of guidelines in favor of hemodialysis in this case, it may have been helpful for the following reasons. CQ and by extension HCQ are known to interfere with potassium efflux and facilitate insulin release [16]. Hypokalemia and hypoglycemia are both known precipitants of cardiac arrest. Perhaps critical hypokalemia and hypoglycemia were averted by stabilizing plasma levels with hemodialysis. Secondly, in the face of the overwhelming overdose, the “lipid sink” provided by ILE may become saturated and thus abolish the gradient for lipophilic compounds to diffuse out from the intracellular compartment to the extracellular compartment. It is conceivable that the “lipid sink” diffusion gradient was preserved by dialysing Intralipid that had become saturated with HCQ. Extracorporeal membrane oxygenation (ECMO) has also been successfully demonstrated in an isolated case severe HCG toxicity [17]. The use of ECMO was not considered in this case.

Intravenous lipid emulsion is commercially available and termed Intralipid for treatment of local anesthetic systemic toxicity (LAST). Early basic science research by Weinberg et al. established that lipid emulsion successfully resuscitated rats and dogs from bupivacaine induced cardiac arrest [18, 19]. This work described the “lipid sink” theory of ILE, whereas ILE may act as an expanded lipid reservoir to sequester lipophilic bupivacaine away from cardiac myocytes [18]. Rosenblatt et al. described the first use of ILE for the treatment of cardiac arrest after bupivacaine and mepivacaine overdose [20]. Subsequently, Litz et al. reported the recovery of a perfusing cardiac rhythm with ILE after prolonged asystolic arrest following a ropivacaine overdose after axillary plexus block [21]. Given these promising developments, the role of ILE was expanded from the treatment of LAST to other toxic overdoses [17, 22]. Similar to bupivacaine, HCQ is lipophilic with a large volume of distribution [23] and blocks sodium channel function [24]. Experience with HCQ overdose is limited as there are few published case reports in the literature. Thus, resuscitation of HCQ overdose is managed similar to CQ overdose given their structural and toxidromal similarities [1]. A recent case report describes two cases of HCQ overdose in which ILE was utilized in the resuscitation [25]. Despite the standard resuscitation of these patients with sodium bicarbonate and diazepam, both died despite ILE infusion. The first case was a mixed overdose of HCQ and CQ in which the patient developed torsades des pointes that failed to respond to single bolus dose of 100 mL of 20% Intralipid. The second patient suffered from a cardiac arrest following a 20 mg HCQ overdose with the return of spontaneous circulation (ROSC) after cardiopulmonary resuscitation (CPR) for 5 minutes. A bolus dose of 100 mL of 10 Intralipid was given followed by 400 mL over 30 minutes. The patient subsequently developed wide complex tachycardia and cardiac arrest with ROSC after 25 minutes of CPR. Despite these interventions, the patient suffered another arrest and died. In both cases, the authors did not institute hemodialysis.

## 4. Conclusion

In conclusion, HCQ poisoning is rare but serious because of its rapid progression to life-threatening symptoms. Hemodynamic support, gastric decontamination, electrolyte monitoring and replacement, and management of arrhythmias are the mainstays of treatment. The combined role of dialysis and ILE in the setting of massive HCQ overdose may improve outcomes by extending the “lipid sink” effect of ILE and normalizing electrolyte concentrations.
